# Synchronous retroperitoneal and mesenteric dedifferentiated liposarcoma: case report of a challenging rare disease and literature review

**DOI:** 10.1093/jscr/rjag744

**Published:** 2026-08-26

**Authors:** Mauro Giambusso, Paolo Di Mattia, Giorgio Maria Paolo Graziano, Alessio Licciardello, Danilo Corrado Centonze

**Affiliations:** Department of Surgery, General Surgery Operative Unit, Umberto I Hospital, Contrada Ferrante, 94100 Enna, Italy; Department of Medicine and Surgery, University of Enna “Kore”, Contrada Ferrante, 94100 Enna, Italy; Department of Medicine and Surgery, University of Enna “Kore”, Contrada Ferrante, 94100 Enna, Italy; Department of Surgery, General Surgery Operative Unit, Umberto I Hospital, Contrada Ferrante, 94100 Enna, Italy; Department of Surgery, General Surgery Operative Unit, Umberto I Hospital, Contrada Ferrante, 94100 Enna, Italy

**Keywords:** liposarcoma, dedifferentiated, retroperitoneal, mesenteric, synchronous

## Abstract

Dedifferentiated liposarcoma (DDL) is a rare malignant mesenchymal tumor affecting predominantly the retroperitoneum. Complete surgical excision following early diagnosis is crucial in these patients due to a high rate of local recurrence and metastasis. We report a rare case of synchronous retroperitoneal and mesenteric DDL presenting both a diagnostic and therapeutic challenge.

## Introduction

Liposarcomas account for around 20% of soft tissue sarcomas [1], thus representing the most common ones, with an incidence of ~1 case per 100 000 people per year. They most commonly arise in the extremities [2] or retroperitoneum [3] of adults, but all body areas can be affected. Presenting symptoms can often be vague and non-specific mostly for patients with retroperitoneal disease, whereas disease in the extremities will often present as a palpable mass. Collectively, histological subtypes include well-differentiated liposarcoma (WDL), dedifferentiated liposarcoma (DDL), myxoid liposarcoma (ML), and the rare pleomorphic liposarcoma (PL) [4]. WDLs [5] are the most common and least aggressive forms, mainly affecting the extremities. DDL [6] may be the result of WDL dedifferentiation, with more aggressive behavior and worse outcome due to a higher rate of local recurrence and the potential to metastasize, predominantly to the lungs. Surgical excision is the treatment of choice for these two subtypes. MLs [7], which mainly affect the extremities of adults at a younger age than the other variants, are typically more responsive to chemo- and radiotherapy. PL [8], the rarest form, is markedly aggressive. It mostly affects the extremities and may have a higher potential for local recurrence and metastasis than DDL, with reduced chemo- and radiosensitivity.

We aim to report a case of synchronous retroperitoneal and mesenteric DDL not only because of the rarity of their coexistence but also for the challenge it posed in terms of diagnosis and management.

## Case report

**Figure 1:**
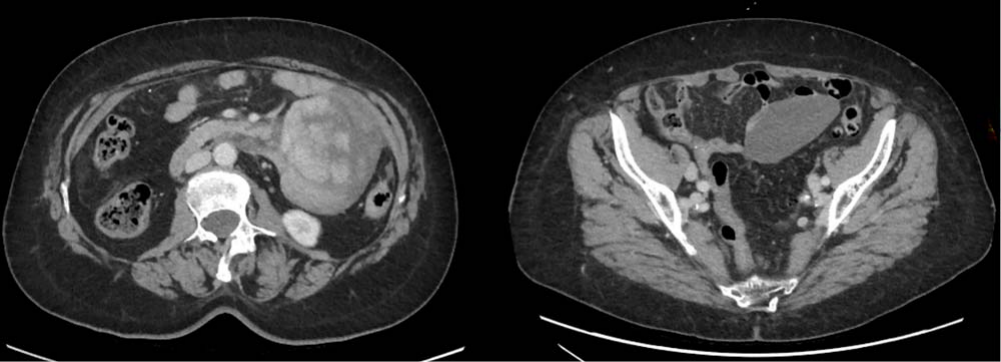
Axial CT-scan: the retroperitoneal liposarcoma on the left and the mesenteric liposarcoma on the right.

**Figure 2:**
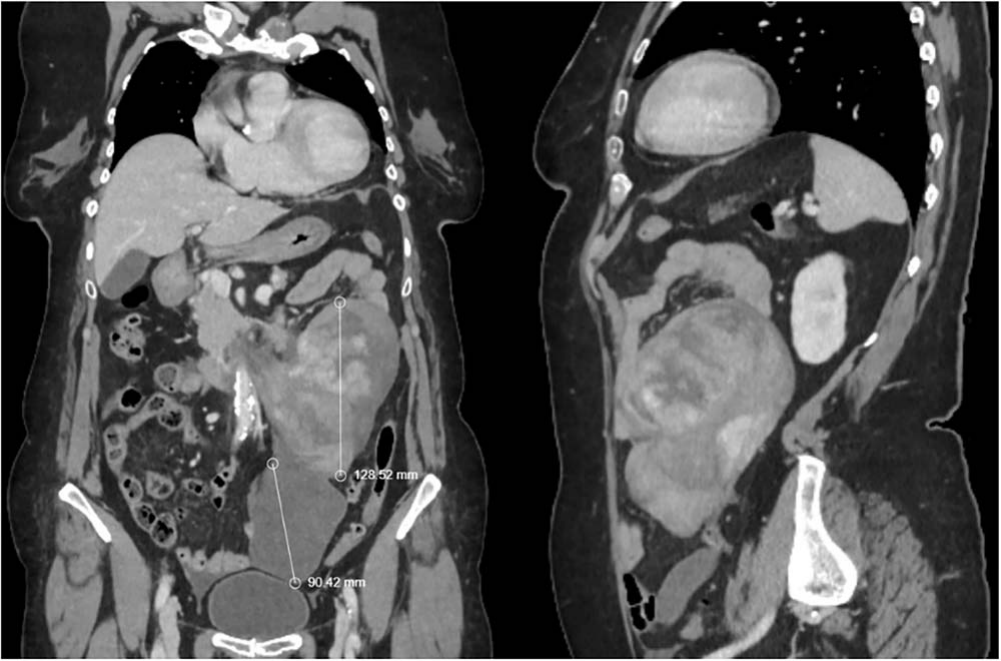
On the left, coronal CT-scan: at the top the retroperitoneal liposarcoma, below the mesenteric one, along with the corresponding maximum diameters; on the right, sagittal CT-scan: from top to bottom the retroperitoneal and mesenteric liposarcoma.

**Figure 3:**
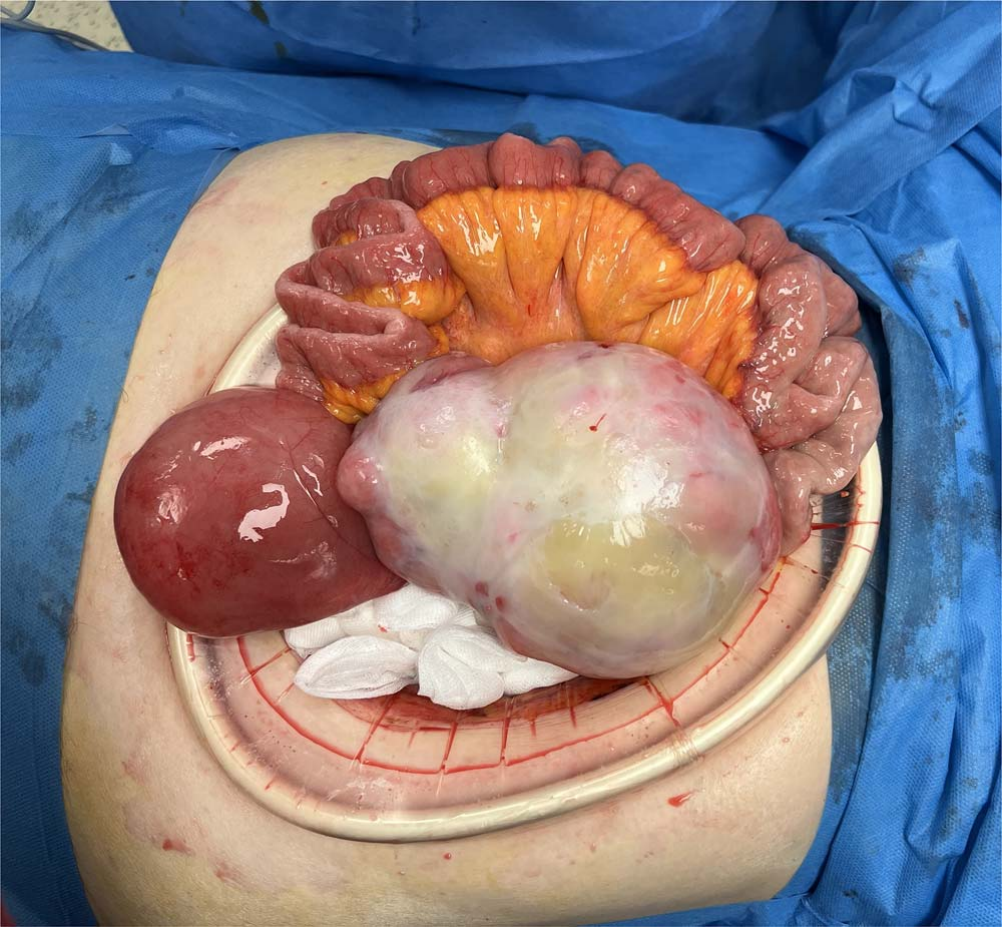
Intraoperative image showing the two synchronous liposarcomas: the mesenteric liposarcoma on the left and the retroperitoneal one on the right.

**Figure 4:**
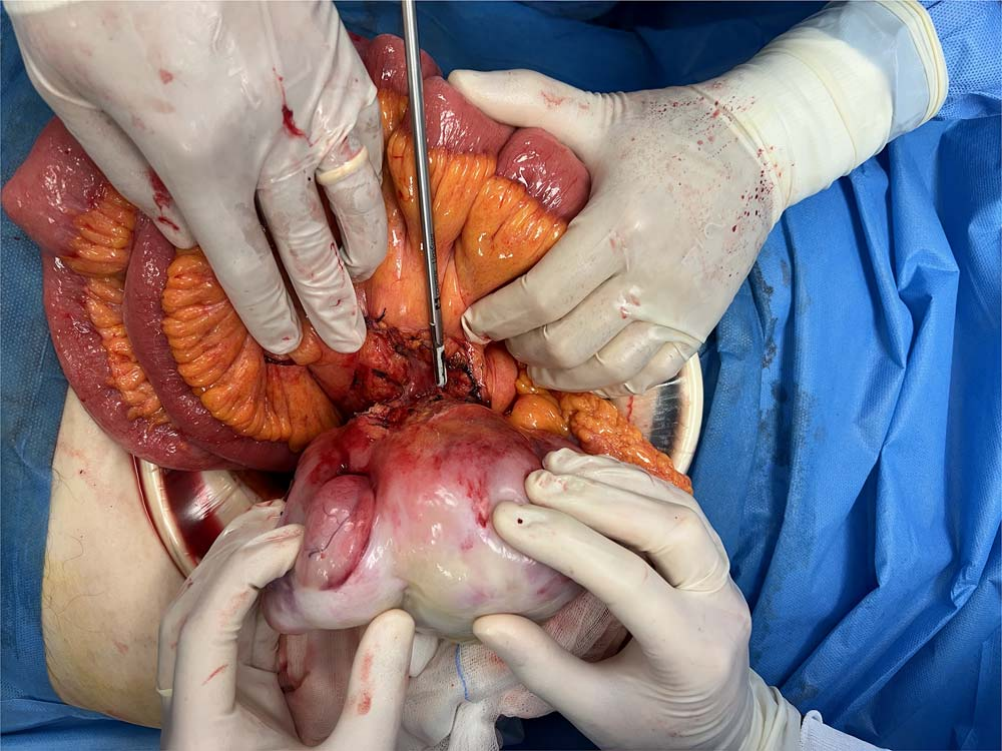
Intraoperative image showing the dissection of the retroperitoneal liposarcoma originating from the third and fourth portions of the duodenum.

**Figure 5:**
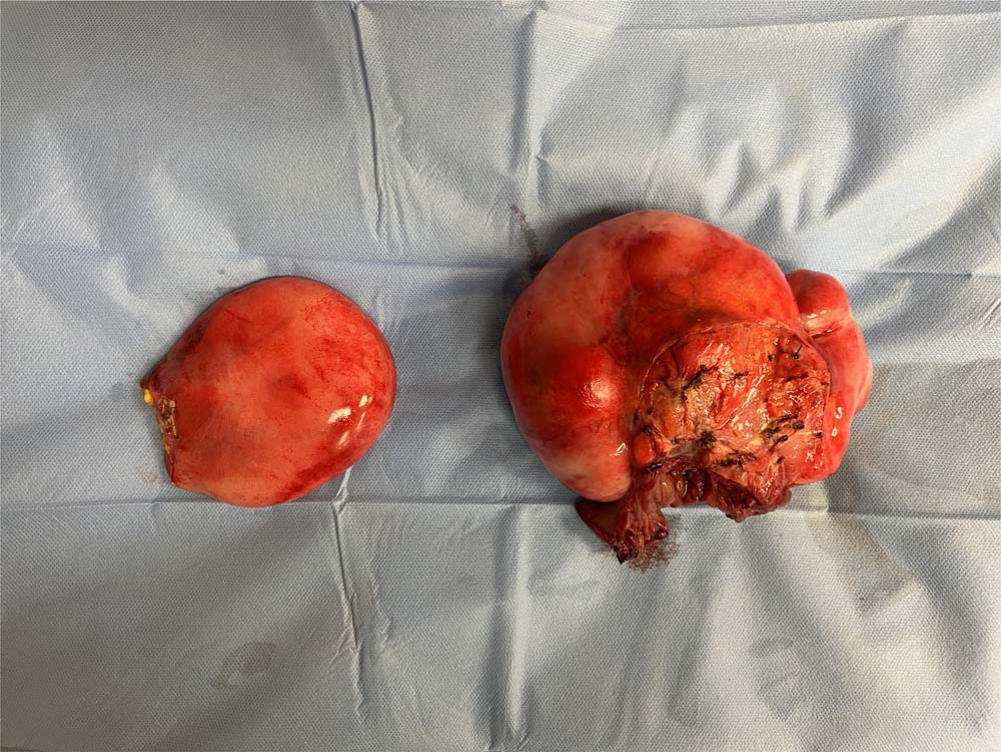
Surgical specimens: on the left the mesenteric liposarcoma, on the right the retroperitoneal liposarcoma.

A 75-year-old female patient undergoing follow-up for chronic kidney disease was found to have a large, previously undetected, mass during abdominal computed tomography (CT)-scan (Figs 1 and 2). The exam showed a massive oval capsular formation of about 13 cm maximum diameter with contrast enhancement encompassing the last duodenal portion. A second cystic neoformation with 9 cm maximum diameter was also found inferiorly and contiguous to the previous one. The patient was in good general condition. The reported symptoms were mild sense of weight and abdominal tension. Comorbidities included arterial hypertension and chronic renal failure. Blood tests were unremarkable. A digestive echoendoscopy was additionally performed, showing a coarse solid hypoechogenic neoformation of about 10 cm maximum diameter in the absence of cleavage planes with the third and fourth portions of duodenum. At explorative laparotomy two distinct tumors were found. The largest was pearly in appearance with fibro-elastic consistency, measuring ~17 cm in maximum diameter, arising from the III and IV segments of the duodenum and adherent to the lower margin of the pancreas. The other one showed a translucent capsule, a gelatinous and soft consistency, measuring up to ~11 cm, with pedicle arising from the root of the mesentery (Fig. 3). Surgical excision of the two distinct neoformations “en-bloc” with duodenal third and fourth portions, the first loop of the jejunum and a small portion of the pancreatic lower margin and uncus due to local infiltration was performed, in order to obtain disease-free margins (Fig. 4). Following duodenal diverticulization, a Roux-en-Y gastrointestinal reconstruction was performed. Although they differ morphologically, histological findings of both surgical specimens (Fig. 5) showed on immunohistochemical profile positivity for murine double minute 2 (MDM2) protein, pathognomonic of DDLs. The patient was discharged on postoperative Day 7 after a short postoperative stay in the intensive care unit, in the absence of complications. Local recurrence detected by follow-up CT-scan occurred 6 months after surgery. Reoperation was not recommended due to the significant abdominal spread and the associated high risk of further recurrence. Palliative chemotherapy was suggested, but the patient declined. Death ensued ~1 year after.

## Discussion

DDL is often the result of the degeneration of preexisting WDL. It generally affects adults and most frequently arises in the retroperitoneum; other sites are far rarer [9].

In this report we present an extremely rare case of synchronous retroperitoneal and peritoneal mesenteric DDL. The mesenteric site has been reported in very few cases in the literature, as shown in the experience of Ahire *et al*. [10].

Diagnosing these conditions is not straightforward, particularly when they are located in the abdomen, as they often grow silently. The radiological diagnostic gold standard for retroperitoneal and intra-abdominal DDL is a contrast-enhanced CT-scan, while magnetic resonance imaging is the preferred method in cases affecting the extremities [11]. Nevertheless, imaging is also essential to assess the relationship with the surrounding vascular and non-vascular structures, in order to determine whether resection can be safely and radically performed.

Indeed, the aim of treatment for DDL remains the complete excision of the tumor, with the goal of achieving an R0 resection due to the risk of local recurrence of ~40%, as well as the risk of metastasis in 20%–30% of cases. In their retrospective study, Dehner *et al*. [12] demonstrated that the presence of disease at the resection margin was significantly associated with shorter local recurrence-free survival, highlighting it as an important prognostic factor. Nevertheless, when surgical radicality cannot be achieved, the use of systemic therapies as neoadjuvant or adjuvant treatment may be considered in order to reduce the risk of recurrence [13].

Systemic therapies as first-line treatment remain limited in clinical practice due to the poor response of this condition. Chemotherapy and radiotherapy are generally reserved for phenotypes as the myxoid or PL, where they seem most effective, especially for myxoids [14]. This is probably due to the immunohistochemical differences between the various subtypes of liposarcoma. In our case, overexpression of MDM2 oncogene caused by mutations on chromosome 12q13–15, pathognomonic of DDL subtype, confirmed the diagnosis. Positivity of vimentin, typically expressed in mesenchymal cells, and p16, extremely sensitive in malignant mesenchymal tumors, was also detected. The focal presence of S100, a protein diffusely positive in WDL, is another common finding indicating the coexistence of well-differentiated low-grade and dedifferentiated high-grade areas of liposarcoma. This suggests how the development of DDL may be the result of a sudden degeneration of the differentiated component.

Although surgical excision symbolizes the gold standard of treatment, in cases of advanced unresectable or metastatic liposarcoma, chemotherapy remains the only alternative.

Anthracyclines represent the first-line treatment, while trabectedin and eribulin are effective second-line treatments [15]. Novel targeted therapies such as MDM2 antagonists and cyclin-dependent kinase 4 inhibitors, which target proteins that are typically overexpressed in DDL, seem to be promising pharmacological options [16].

In conclusion, abdominal DDLs are rare tumors with worst outcome due to high risk of local recurrence and metastasis. Early diagnosis and the achievement of radical surgical resection enable better long-term outcomes in these patients. Pharmacotherapy may offer an alternative in advanced cases where curative treatment is not feasible, with emerging targeted therapies looking encouraging.
